# Chaperone-mediated native folding of a β-scorpion toxin in the periplasm of *Escherichia coli*^☆^

**DOI:** 10.1016/j.bbagen.2013.08.021

**Published:** 2014-01

**Authors:** A.O. O'Reilly, A.R. Cole, J.L.S. Lopes, A. Lampert, B.A. Wallace

**Affiliations:** a Department of Physiology and Pathophysiology, Friedrich-Alexander Universität Erlangen-Nürnberg, Universitätsstraße 17, 91054 Erlangen, Germany; b Institute of Structural and Molecular Biology, Birkbeck College, University of London, London, UK

**Keywords:** Chaperones, Disulfide-linked toxin, Protein folding, Protein expression, Crystal structure, Synchrotron radiation circular dichroism spectroscopy

## Abstract

**Background:**

Animal neurotoxin peptides are valuable probes for investigating ion channel structure/function relationships and represent lead compounds for novel therapeutics and insecticides. However, misfolding and aggregation are common outcomes when toxins containing multiple disulfides are expressed in bacteria.

**Methods:**

The β-scorpion peptide toxin Bj-xtrIT from *Hottentotta judaica* and four chaperone enzymes (DsbA, DsbC, SurA and FkpA) were co-secreted into the oxidizing environment of the *Escherichia coli* periplasm. Expressed Bj-xtrIT was purified and analyzed by HPLC and FPLC chromatography. Its thermostability was assessed using synchrotron radiation circular dichroism spectroscopy and its crystal structure was determined.

**Results:**

Western blot analysis showed that robust expression was only achieved when cells co-expressed the chaperones. The purified samples were homogenous and monodisperse and the protein was thermostable. The crystal structure of the recombinant toxin confirmed that it adopts the native disulfide connectivity and fold.

**Conclusions:**

The chaperones enabled correct folding of the four-disulfide-bridged Bj-xtrIT toxin. There was no apparent sub-population of misfolded Bj-xtrIT, which attests to the effectiveness of this expression method.

**General significance:**

We report the first example of a disulfide-linked scorpion toxin natively folded during bacterial expression. This method eliminates downstream processing steps such as oxidative refolding or cleavage of a fusion-carrier and therefore enables efficient production of insecticidal Bj-xtrIT. Periplasmic chaperone activity may produce native folding of other extensively disulfide-reticulated proteins including animal neurotoxins. This work is therefore relevant to venomics and studies of a wide range of channels and receptors.

## Highlights

• Novel method for producing natively folded disulfide linked toxins
• Co-expression in periplasmic space with chaperones
• Crystal structure has the same structure/disulfide links as toxin from natural source.
• Synchrotron radiation circular dichroism spectroscopy shows thermal stability.
• Potential uses in studies of channel and receptor structure/function relationships

## 1 Introduction

A diverse array of animals including scorpions, spiders, conus snails and sea anemones produce venom replete with small peptide neurotoxins to incapacitate prey or predators. Most of these toxins modify the function of ion channels of the nervous, muscular or cardiovascular systems [1]. The toxins display variability in length, sequence, folds and molecular targets but share a common structural property in that in each case a network of disulfide bridges stabilizes their respective fold [2]. This extensive reticulation establishes the tertiary structure that presents the bioactive surface as well as conferring significant thermostability [3] and resistance to proteolysis.

**Fig. 1:**
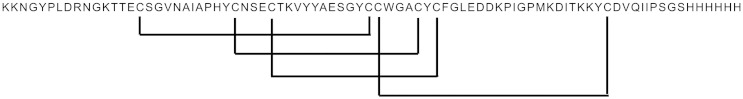
Sequence and disulfide-connectivity map of Bj-xtrIT-(His_6_).

β-Scorpion toxins are polypeptides of 60–76 residues stabilized by four disulfide bridges [4]. They bind with nanomolar affinity to an extracellular receptor (site 4) of voltage-gated sodium channels [5]. β-Toxins induce a hyperpolarizing shift of the voltage-dependence of activation via a voltage-sensor trapping mechanism, whereby toxin-mediated interactions stabilize a persistently-activated conformation of the channel domain II voltage sensor [6,7]. The extensively-characterized Bj-xtrIT from *Hottentotta judaica* (Fig. 1) is a member of the ‘excitatory’ (inducing a spastic paralysis) anti-insect β-toxins [8–11] and its structure was the first of this class to be solved by X-ray crystallography [11].

The low cost, simplicity, speed-of-growth and wide-spread availability of bacterial-culturing facilities have made *Escherichia coli* the host organism of choice for recombinant protein production. However, the reducing environment of the *E. coli* cytoplasm can hinder disulfide-bond formation and render cysteine-containing proteins prone to misfolding and aggregation [12]. Previous reports of Bj-xtrIT prepared from *E. coli* have required the solubilization of misfolded toxin from inclusion bodies under denaturing and reducing conditions [8]. Other reports of recombinant scorpion toxins circumvent this step by using a fusion chimera to rescue protein from incorporation into inclusion bodies [13,14]. However, this latter approach still requires an additional processing step to cleave the fusion partner and yield the mature toxin. Further downstream processing typically involves in vitro oxidative refolding using reagents such as reduced and oxidized forms of glutathione to produce disulfide-bridge shuffling with the goal of obtaining native connectivity. A post-folding purification step is often required to isolate the correct structural isomer from the misfolded ensemble. Indeed, identifying which elution fraction from a chromatography step corresponds to the correctly-folded isomer can require arduous bio-assay, electrophysiological or structural studies.

In this study we show that the Bj-xtrIT β-scorpion toxin can be expressed in its natively-folded conformation by secretion of the polypeptide into the oxidizing environment of the *E. coli* periplasm [15]. However, we found that the toxin could not be over-expressed and purified when secreted alone. Instead, periplasmic co-secretion of the four bacterial chaperone proteins DsbC, DsbA, SurA and FkpA encoded by the pTUM4 plasmid [16] enabled robust toxin over-expression. Chromatographic analyses demonstrated the homogeneity and monodispersity of the purified sample and synchrotron radiation circular dichroism spectroscopy confirmed that the recombinant toxin was thermostable. Finally, we crystallized and solved the structure of recombinant Bj-xtrIT to confirm that it indeed adopts the correctly-reticulated native fold.

## 2 Material and methods

### 2.1 Molecular biology

**Supplementary Fig. 1:**
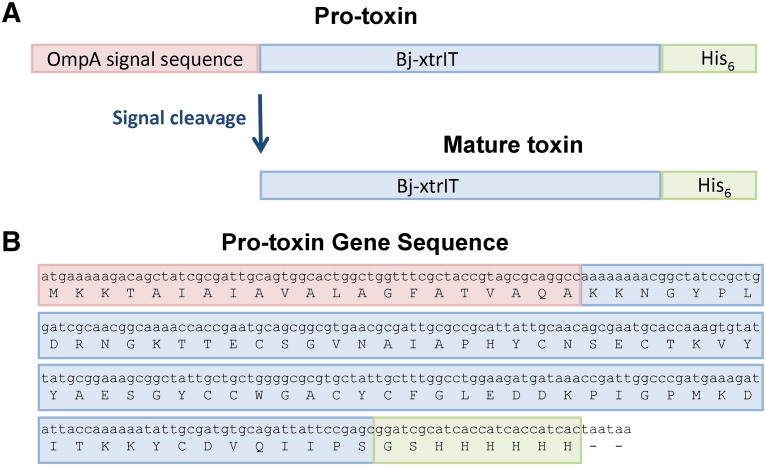
(A) Schematic diagram showing processing of the protoxin to the mature forms of Bj-xtrIT-(His_6_) by the signal peptidase. (B) DNA and translated protein sequences of the Bj-xtrIT-(His_6_) pro-toxin gene sub-cloned into the pASK-IBA32 vector. Stop codons are represented by ‘-’. Sequences are shaded according to the diagram in part (A).

A gene encoding the Bj-xtrIT sequence (Swiss-Prot accession code P56637) with a bacterial codon bias and a C-terminal hexa-histidine tag was synthesized by Gene Oracle (California, USA). The gene was PCR-amplified using the primers 5′-GGTTTCGCTACCGTAGCGCAGGCCAAAAAAAACGGCTATCCGCTGGATCGTAATGGTA-3′ and 5′-CAAGCTTATTAGTGATGGTGATGGTGATGCGATCCGCTCGGAATAATCTGCACGT-3′ and sub-cloned into the pASK-IBA32 expression vector (IBA GmbH, Göttingen, Germany) using the overlap extension PCR cloning method [17] with Phusion polymerase (New England Biolabs). DH5α chemically-competent *E. coli* cell (New England Biolabs) transformants were plated overnight at 37 °C on 100 μg/ml ampicillin (AMP) agar plates. Plasmid-DNA isolated from single colonies was sequenced to verify that the construct had the correct sequence (Supplementary Fig. 1).

### 2.2 Expression and periplasm extraction

Chemically-competent BL21 (DE3) *E. coli* cells (Invitrogen) were transformed with the pTUM4 plasmid (generously provided by A. Skerra of Technische Universität München, Freising-Weihenstephan, Germany) and plated on agar containing 25 μg/ml chloramphenicol (CAM) overnight at 37 °C. A single colony was used for the growth and preparation of 200 μl aliquots of cells, which were made chemically competent using the magnesium/calcium chloride method as described [18]. These pTUM4 transformants were further transformed with pASK-IBA32 plasmid encoding the Bj-xtrIT-(His_6_) gene and plated on agar with 100 μg/ml AMP plus 25 μg/ml CAM overnight at 37 °C. Alternatively, chemically-competent BL21 *E. coli* cells not harboring the pTUM4 plasmid were transformed with the pASK-IBA32/Bj-xtrIT-(His_6_) and plated on AMP plates.

A single colony was used to inoculate LB medium (Melford) containing 100 μg/ml AMP and, for cells harboring pTUM4, additionally 25 μg/ml CAM. 1 l of antibiotic-supplemented LB medium was inoculated with 10 ml of pre-culture (grown overnight at 37 °C) and shaken at 200 rpm at 37 °C until an OD_600_ = 0.5 was reached. Toxin expression was induced using 200 μg/l anhydrotetracycline (Fluka) and the culture was grown at 26 °C for 8 h. Cells were harvested at 4400 g for 15 min at 4 °C. The supernatant was discarded and the pellet was placed on ice and resuspended in 10 ml of ice-cold periplasm-extraction buffer: 500 mM sucrose, 1 mM EDTA, 100 mM Tris–HCl; pH 8. Following 30 min incubation on ice, spheroplasts were spun down at 5000 *g* for 15 min at 4 °C and the supernatant was further centrifuged at 27000 *g* to yield the clarified periplasm fraction.

### 2.3 Toxin purification

Bj-xtrIT-(His_6_) toxin was purified by immobilized-metal affinity chromatography (IMAC) using a 5 ml HisTrap HP nickel column (GE Healthcare). The column was pre-equilibrated with 20 mM Tris; pH 7.8, 150 ml NaCl, 20 mM imidazole. Periplasm extract was filtered (0.2 μm filter) and passed through the column overnight at 4 °C using a peristaltic pump. An Äkta purifier (GE Healthcare) was used to apply a linear gradient of imidazole (20 mM to 500 mM) to wash and elute the toxin. The elution was concentrated using a Vivaspin (Sartorius) with a 5 kDa molecular weight cut-off. Size exclusion chromatography using a Superdex 75 10∕300 (GE Healthcare) column pre-equilibrated with 20 mM Tris; pH 7.8, 150 ml NaCl and operated with a flow-rate of 0.5 ml/min provided the final preparative step. A 20 μl aliquot of the IMAC elution was also applied to a Jupiter C18 5 μm 250 mm × 4.6 mm reverse-phase HPLC analytical column (Phenomenex) and eluted using a 5% to 95% acetonitrile gradient.

### 2.4 SDS-PAGE and Western blot analysis

Samples were prepared for SDS-PAGE by heating at 90 °C for 10 min with 2.5% (w/v) SDS, 2% (v/v) β-mercaptoethanol. 10 μl aliquots were electrophoresed for 25 min at 200 mV using a 4–12% NuPAGE Bis-Tris gel (Invitrogen); molecular weight markers were Ultra-Low Range (Sigma). The gel was either stained with SimplyBlue SafeStain (Invitrogen) or used with an iBlot system (Invitrogen) to transfer protein to a nitrocellulose membrane for Western blotting. The blot was probed with an anti-His (C-term) antibody conjugated with alkaline phosphatase (Invitrogen) and developed using a Sigmafast 5-bromo-4-chloro-3-indolyl phosphate/nitro blue tetrazolium tablet (Sigma).

### 2.5 Synchrotron radiation circular dichroism (SRCD) spectroscopy

Bj-xtrIT-(His_6_) toxin purified by IMAC and size-exclusion chromatography was prepared for thermal denaturation studies by buffer-exchanging the sample into 20 mM sodium phosphate; pH 7.8, 5% glycerol using a 5 ml HiTrap Desalting column (GE Healthcare). The SRCD spectrum of Bj-xtrIT-(His_6_) toxin (3.3 mg/ml) was collected using a 0.0015 cm pathlength demountable quartz cell (Hellma Scientific) at beamline CD1 at the Institute for Storage Ring Facilities (Aarhus, Denmark). Data were collected over the wavelength range of 270 to 175 nm with a 1 nm interval and 2 s dwell time. Protein was incubated at temperatures ranging from 25 to 85 °C, in 5 °C steps allowing 5 min equilibration at each temperature. At each temperature point three scans of either the sample or baseline (buffer without protein) were obtained. The first and last spectra were compared to ensure that equilibrium had been reached prior to spectral data collection. The three scans were averaged and the averaged baseline spectrum was subtracted from the averaged sample spectrum, smoothed with a Savitzky–Golay filter, calibrated versus a camphorsulfonic acid standard, and scaled to Delta Epsilon units using a mean residue weight of 113. All data processing was done using CDTools software [19]. Secondary structure analyses used the Dichroweb server [20], with database SP175 [21] and the ContinLL [22] algorithm.

### 2.6 X-ray crystallography

Purified Bj-xtrIT-(His_6_) toxin at a concentration of 10 mg/ml was mixed 1:1 (v/v) with 0.2 M NaCl, 0.1 M Bis-Tris; pH 5.5, 29% PEG 3350. 2 μl hanging drops were equilibrated at 16 °C against 500 μl of the crystallization solution. Crystals appeared within one week and were prepared for cryo-freezing by immersing in crystallization solution supplemented with 35% PEG 3350.

Data were collected on the Proxima1 beamline at the Soleil Synchrotron, France to a maximum resolution of 1.7 Å. Integration and scaling of the data were carried out using XDS [23] with the SCALA program from CCP4 [24] being used for further data analysis. Molecular replacement was carried out using the native toxin structure (PDB ID: 1BCG) with the PHASER package [25], as implemented in CCP4. Refinement was carried out using the BUSTER suite [26]. Structure analysis and building were carried out using COOT [27]. A figure was produced using PyMOL (DeLano Scientific, CA, U.S.A.). Coordinates of Bj-xtrIT-(His_6_) have been deposited in the Protein Data Bank (PDB ID: 4KYP).

## 3 Results

### 3.1 Periplasmic co-secretion of chaperones enables Bj-xtrIT-(His_6_) over-expression

**Fig. 2:**
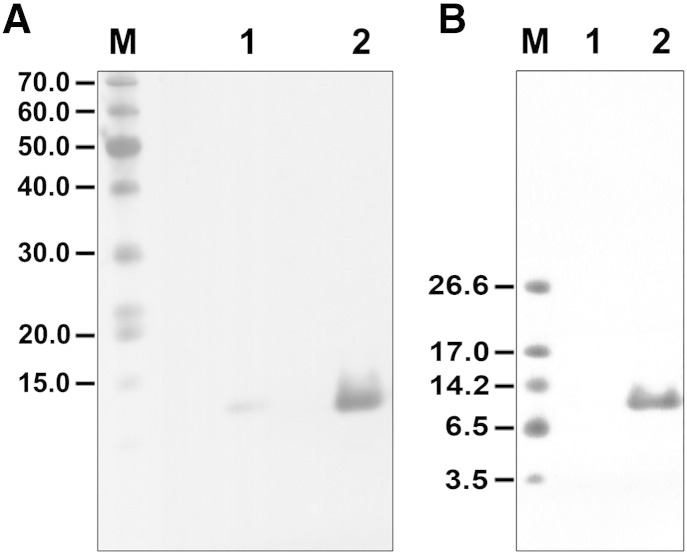
Expression and purification of Bj-xtrIT-(His_6_). (A) Western blot analysis: lane M, molecular weight markers; lane 1, periplasmic cell extract of *E. coli* BL21/pASK-IBA32; and lane 2, periplasmic cell extract of *E. coli* BL21/pASK-IBA32/pTUM4. (B) SDS-PAGE analysis following IMAC purification: lane M, molecular weight markers; lane 1, periplasmic cell extract of *E. coli* BL21/pASK-IBA32; and lane 2, periplasmic cell extract of *E. coli* BL21/pASK-IBA32/pTUM4.

To produce natively-folded scorpion toxin directly from a bacterial expression system, we sub-cloned the Bj-xtrIT-(His_6_) gene into the pASK-IBA expression vector, thereby introducing an OmpA secretion signal at the toxin N-terminus. This sequence directs polypeptide secretion via the Sec pathway in the oxidative compartment of the *E. coli* periplasm. The signal is subsequently cleaved by an endopeptidase, producing the target gene product without an N-terminal adduct. The level of Bj-xtrIT-(His_6_) expression was extremely low (Fig. 2A; lane 1). We attempted to use immobilized-metal affinity chromatography to isolate soluble toxin from the periplasmic extract in order to obtain a more concentrated solution of protein for visualization using SDS-PAGE. The toxin in its reduced form should migrate with a molecular mass of ~ 9.4 kDa but no band was observed on a 4–12% SDS-PAGE gel (Fig. 2B; lane 1). These results are representative of Bj-xtrIT-(His_6_) expression trials using three individual *E. coli* transformants and suggest that the secreted protein may be inherently labile.

Then cells were co-transfected with the pTUM4 plasmid [16] to determine the effect of chaperone activity on Bj-xtrIT-(His_6_) expression. This helper vector encodes genes for the DsbA and DsbC members of the thioredoxin superfamily [28] and genes for SurA and FkpA peptidyl-prolyl *cis*/*trans*-isomerases. These enzymes are all expressed under a constitutive promoter and secreted into the periplasm, where they are available to operate on newly-secreted toxin. Under identical conditions of cell growth and genetic induction, there was significant Bj-xtrIT-(His_6_) expression (Fig. 2A; lane 2) from cells harboring pTUM4. In addition, protein could be purified to homogeneity (Fig. 2B; lane 2) with a final yield of ~ 0.3 mg/l culture. Hence, the presence of chaperones in the periplasm promoted toxin expression and yielded suitable amounts of soluble protein for subsequent biochemical and structural characterization.

### 3.2 Bj-xtrIT-(His_6_) homogeneity

**Fig. 3:**
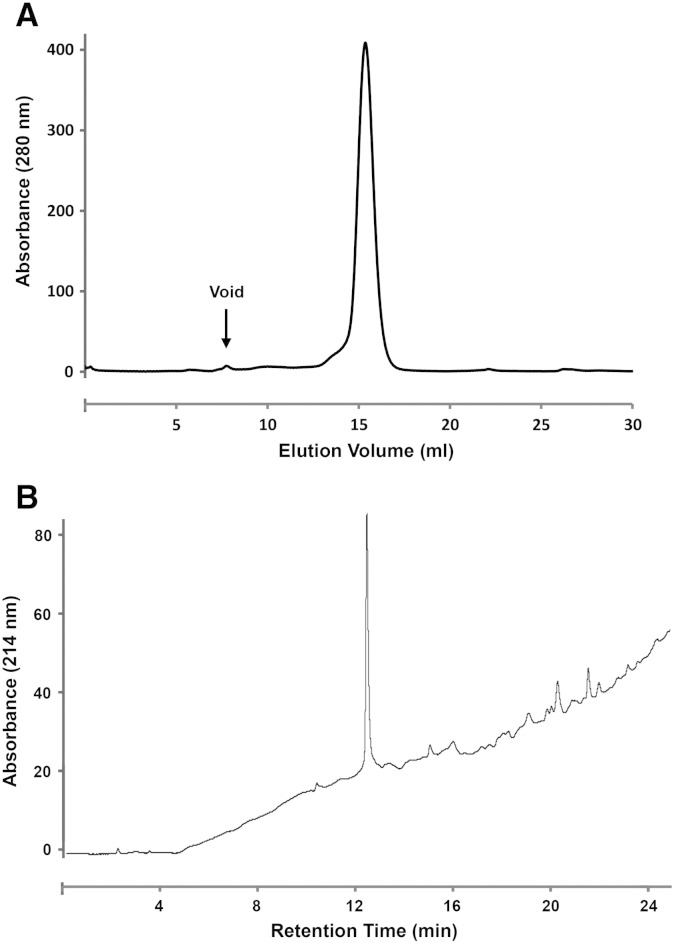
Chromatography analysis of Bj-xtrIT-(His_6_) IMAC eluate. (A) Size-exclusion HPLC. (B) Analytical reverse-phase FPLC.

Bj-xtrIT-(His_6_) from pTUM4-plasmid transformants was purified by IMAC (Fig. 2B) and analyzed by size exclusion chromatography. The elution profile shows a single symmetrical peak that was retained longer than the void volume (Fig. 3A). The IMAC eluate was also examined using analytical RP-HPLC and the elution profile also exhibited a single major peak (Fig. 3B). Together these results indicate that the purified protein was soluble, homogenous and mono-disperse.

### 3.3 Bj-xtrIT-(His_6_) thermostability

**Fig. 4:**
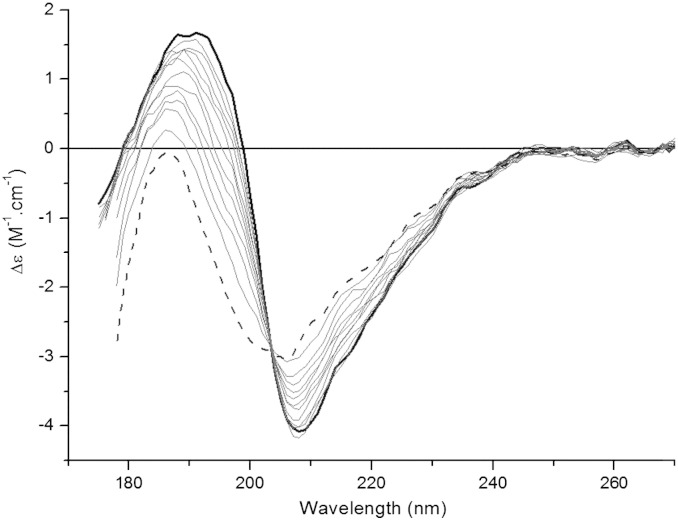
Thermal denaturation of Bj-xtrIT-(His_6_) monitored by SRCD spectroscopy. Toxin was incubated over the temperature range from 25 °C (bold line) to 85 °C (dash line) in 5 °C increments (intermediate curves are in gray).

The SRCD spectrum of the toxin at 25 °C displays a large negative peak at 207 nm (Fig. 4), which matches the previously-reported CD spectrum of Bj-xtrIT [9], plus an additional positive peak at 190 nm with a shoulder at 185 nm (not seen previously as that study had used conventional CD spectroscopy). The estimate of the secondary structural content from the SRCD data was 25% α-helix, 23% β-sheet and 52% other structures, very similar to the content determined from the crystal structure of Bj-xtrIT (PDB ID: 1BCG), calculated using the 2Struc server (26% α-helix, 20% β-sheet and 54% other) [29].

The thermostability of Bj-xtrIT-(His_6_) was then investigated using SRCD spectroscopy, which represents the first application of this sensitive technique to this class of protein. Using SRCD spectroscopy as opposed to CD spectroscopy enabled a more accurate observation of the peak at ~ 186 nm, which is indicative of a denatured protein. The entire SRCD spectrum was monitored from 25 to 85 °C as the temperature was increased in 5 °C steps. Discrete but relatively small differences from the original spectrum were observed with increasing temperature, with a reduction of the intensity of the peak at 207 nm being the most apparent change. Nevertheless, even at very high temperatures the SRCD spectra did not resemble one typical for a denatured protein. The estimate of the secondary structural content at 85 °C was 13% α-helix, 29% β-sheet and 58% other structures, which is still similar to the starting structure. Localized denaturation of the α-helical regions could account for the temperature-dependent changes observed by SRCD signal, but overall the protein displayed significant thermostability that is consistent with a correctly-reticulated toxin structure.

### 3.4 Recombinant Bj-xtrIT adopts the native fold

**Fig. 5:**
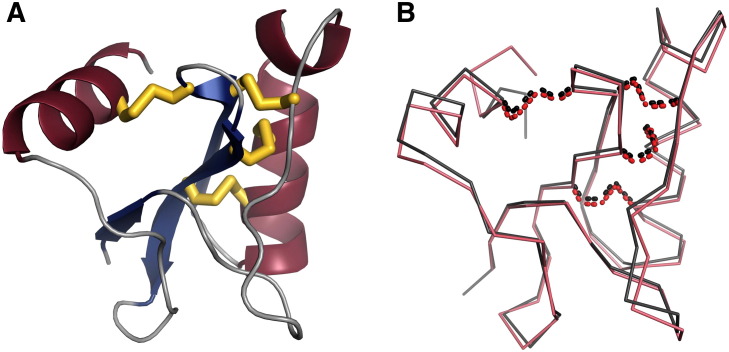
Crystal structures. (A) 1.7 Å crystal structure of Bj-xtrIT-(His_6_) (PDB ID: 4KYP) colored by secondary structure (α-helices; red, β-strands; blue; disulfide bridges, yellow). (B) Superposition of Bj-xtrIT-(His_6_) (PDB ID: 4KYP) (pink backbone; disulfide bridges as red dots) and native Bj-xtrIT (PDB ID: 1BCG) (gray backbone; disulfide bridges as black dots).

Crystals of Bj-xtrIT-(His_6_) diffracted to 1.7 Å resolution (Table 1) and were solved by molecular replacement using the previously-reported Bj-xtrIT structure [11]. Recombinant Bj-xtrIT-(His_6_) has both the βαββα-fold and C1–C4, C2–C6, C3–C7, and C5–C8 disulfide connectivity of the native fold (Figs. 1 and 5A). An all-atom structural superimposition of Bj-xtrIT-(His_6_) with the untagged Bj-xtrIT structure [11] (Fig. 5B) shows that there is a root-mean-square deviation (RMSD) of 0.45 Å. The structures differ only in side-chain rotamers for some residues located on the surface of the toxin, reflecting the difference in crystal contacts that the toxin forms under different crystal packing conditions (space groups C222_1_ vs I4_1_22). Nevertheless the backbone atoms and disulfide groups all superimpose and show that recombinant Bj-xtrIT-(His_6_) was expressed with the native conformation.

## 4 Discussion

Bacterial recombinant expression of toxins with multiple disulfide bonds can be a challenging task that requires considerable optimization of conditions. In particular, post-purification in vitro oxidative refolding is often necessary to yield functionally-folded toxin, which may need to be subsequently identified and separated from non-physiological structural isomers. Here we report an expression strategy that produced natively-folded β-scorpion toxin in the *E. coli* bacterium. Over-expression and purification of Bj-xtrIT-(His_6_) were achieved when a suite of chaperones was co-secreted into the periplasm. With biochemical, biophysical and structural characterization, we demonstrated that the toxin adopts a single conformation with the native fold.

Periplasmic secretion using *E. coli* has proved a successful expression strategy for the functional folding of many disulfide-rich proteins e.g. antibody fragments [30]. However, to date there have been no reports of the successful application of this strategy for scorpion toxins lacking a fusion carrier [31]. Indeed, we found that toxin over-expression could not be achieved solely by secreting Bj-xtrIT into the periplasm. β-Toxins contain eight cysteines and different disulfide pairings can theoretically produce 105 structural isomers [32]. Therefore, although the oxidative environment of the periplasm enables disulfide formation, the potential for misfolding through cysteine mispairing is considerable. An additional factor that can compromise folding is *cis–trans* isomerization of proline residues: Bj-xtrIT has five prolines and each adopts the *trans* configuration in the native fold. In the absence of a mechanism for correcting protein misfolding, the toxin may be susceptible to proteolytic degradation and aggregation in the periplasm [33], which could account for the poor expression and our inability to yield the purified product without pTUM4 co-transfection.

The pTUM4 plasmid secretes four bacterial folding-catalysts into the periplasm [16]. DsbA and DsbC are thiol-disulfide oxidoreductases that work in tandem to produce disulfide-bridge shuffling in a polypeptide substrate and permit the stable low-energy conformation of the native fold to be adopted. Specifically, DsbA functions by rapidly transferring a disulfide bond to a substrate to catalyze disulfide bridge formation. DsbC has two thioredoxin domains and performs an isomerization role by breaking unstable disulfides. A covalent complex is formed with the substrate, which allows a second reactive cysteine in the misfolded protein to react and ideally form a more stable disulfide pairing in the released protein [28]. The second category of secreted folding-catalyst is peptidyl-prolyl *cis*/*trans*-isomerases. The main substrate for SurA and FkpA enzymes is prolyl-iminopeptide bonds, although they appear additionally to have a general chaperone activity that promotes protein folding. Successful chaperone-mediated folding of the target protein has the added advantage that cell lysis is suppressed, which has been attributed to the toxic effects of protein aggregation in the periplasm that can occur in the chaperones' absence [16].

There are many advantages of using this chaperone-mediated expression strategy to produce recombinant β-toxins, including the elimination of downstream processing steps of solubilization and refolding from inclusion bodies [8] or cleavage of a fusion carrier [13,14]. With the current method, no such procedures need be undertaken, as the already-folded toxin can be isolated directly from the periplasm; the procedure is further simplified as there is no need for post-folding separation of structural isomers. Periplasmic extraction also functions as a pseudo-purification step as intact cell bodies are separated as spheroplasts, preventing the release of cytoplasmic contaminants including deleterious proteases. Finally, as the immature polypeptide encodes an N-terminal OmpA secretion signal that is subsequently cleaved in the periplasm, the first residue of the mature toxin does not need to be methionine to function as the start codon for transcription initiation, which is the case with directly-expressed polypeptides. In addition, because there appears to be little or no dependence of the cleavage efficiency of the OmpA secretion signal based on the nature of the amino terminal residue of the mature peptide [34] this method should be generally applicable to polypeptides containing any type of N-terminal residue.

The toxin composition of venom from arthropods, mollusks and cnidarians is characteristically diverse. A multitude of sequence variants within each toxin class contributes to this heterogeneity and the number of small, multidisulfide-bridged animal neurotoxins may total several million [35]. These peptides represent valuable probes for investigating structure–function relationships in ion channels and may also be exploitable as novel therapeutics, e.g. a calcium-channel blocking ω-conotoxin [36] has been commercialized as the analgesic ziconotide (Prialt™). Toxins that display specificity for a single molecular target e.g. the pain-associated sodium-channel Nav1.7 isotype [37], are particularly desirable. However, the isolation of these toxins in significant quantities from natural sources is unrealistic when they are present at very low concentrations in the venom. The cloning of toxin genes can offer a solution to this problem, so development of robust methods for functional expression of toxins in bacteria will support the efforts of venomics projects. The report of a spider toxin functionally-expressed in the *E. coli* periplasm (albeit as a fusion protein with DsbC coupled to the N-terminus) [38] supports our suggestion that chaperone-mediated periplasmic expression may be generally applicable for functional expression of members from different toxin classes. However, this work further extends the efficient preparation of such toxins by eliminating cleavage and additional purification steps.

## 5 Conclusion

We report an expression strategy that uses chaperone-mediated folding in the periplasm to produce correctly-folded Bj-xtrIT β-scorpion toxin in *E. coli*. This represents the first reported instance of a disulfide-linked toxin purified directly from a bacterial expression system without the use of a fusion partner, or requiring solubilization of the expressed toxin from inclusion bodies, followed by refolding in vitro. Furthermore it yields a single conformation with the native disulfide connectivity. This expression strategy represents a time- and cost-effective solution that will enable routine toxin production and mutagenesis to aid in the investigation of channel structure–function relationships.

The following is the Supplementary data related to this article.

## Figures and Tables

**Table 1:** Crystallography statistics for PDB ID: 4KYP.

Data processing statistics	
Space group	C222_1_
a, b, c (Å)	41.87, 88.56, 183.58
α, β, γ (°)	90.0, 90.0, 90.0
Resolution (Å)	44–1.7 (1.77–1.7)
Number of reflections used in refinement	26,392
Completeness (%)	44–2.1 (97.9)
	2.1–1.7 (29.6)
I/σ(I)	9.12 (1.4)
R-sym (%)	10.2 (69.5)
CC 1/2	97.9 (40.1)

Refinement statistics	

R cryst	19.93 (21.3)
R free	23.06 (28.0)
Wilson B-factor (Å^2^)	24.04
Bond lengths (Å)	0.010
Bond angles (°)	0.99

Highest resolution shell is shown in parentheses.
